# Diabetes is not a negative prognostic factor for 30-days mortality after surgery for acute type A aortic dissection

**DOI:** 10.1097/XCE.0000000000000306

**Published:** 2024-06-19

**Authors:** Veronica Lorenz, Luigi Muzzi, Eugenio Neri

**Affiliations:** a Cardiac Surgery - Aortic Unit, University of Study of Siena, Siena Italy

**Keywords:** aortic dissection, cardiac surgery, diabetes

## Abstract

**Background:**

The correlation between diabetes and aortic dissection is not fully understood yet, although in literature many studies have suggested that there may be an association between the two conditions. The purpose of this study is to evaluate whether diabetes represents a short- and long-term risk factor for mortality from type A acute aortic dissection.

**Materials and methods:**

A total of 340 patients with the diagnosis of type A acute aortic dissection underwent aortic surgery between January 2002 and March 2023. The sample was divided into 2 cohorts according to the presence of diabetes (*n* = 34) or not (*n* = 306).

**Results:**

The mean age was 66 (±12.4) years and 60.9% were male. The primary endpoint was 30-day mortality. Hospital mortality was 12 (35.3%) for the diabetes group and 70 (22.9%) for nondiabetes group (*P* = 0.098). Overall survival at 10 years was 48.3% [95% confidence interval (CI): 41.6–54.7%], while the 10-year survival for people with diabetes was 29.5% (95% CI: 13.2–47.9%) and for nondiabetes group 50.6% (95% CI: 43.4–57.3%) (Log-rank, *P* = 0.024).

**Conclusion:**

Diabetes was not found to be a risk factor associated with 30-day mortality in patients undergoing surgery for type A acute aortic dissection. It was a risk factor for long-term survival, but this may be related to diabetes complications.

## Introduction

During the last decades the incidence of diabetes mellitus (DM) and acute aortic pathologies have progressively increased [1,2].

Acute type A aortic dissection (TAAD) is a life-threatening condition characterized by a tear in the inner layer of the aorta. Thirty-day mortality is still very high today, and largely depends on the preoperative clinical factors of each individual patient [3,4].

Diabetes, on the other hand, is a chronic metabolic disorder characterized by high blood sugar levels. Patients with diabetes carry a higher risk of cardiovascular diseases (CVDs) and several clinical studies reported that diabetes is negatively correlated with the incidence of TAAD [5,6]. However, it seems to be inversely related to the aortic aneurysm’s rupture. For some authors, insulin resistance plays a causal factor in dissection by affecting nutrient metabolism and the release of inflammatory cytokines [7].

The aim of our study is to evaluate whether diabetes also negatively affects the risk of mortality for aortic dissection.

## Materials and methods

### Patient selection

We conducted a retrospective observational study, evaluating 340 patients undergoing surgery for TAAD. The patients were admitted to the cardiac surgery unit at the Santa Maria alle Scotte University Hospital of Siena between January 2002 and March 2023. Data relating to preoperative, intraoperative and postoperative variables were collected for each patient and an analysis database was built. Patients were divided into two groups according to the presence of diabetes or not. Patients included in the diabetic group were those with a confirmed diagnosis prior to surgery, independently of glycemic control and mean glycated hemoglobin.

Considering the retrospective nature of this study, the Institutional Ethics Review Board (TAAD23547) has given its approval, without the need to have the written informed consent.

### Statistical analysis

The statistical analysis was performed using IBM SPSS Statistics (version 22.0 Inc., Chicago, Illinois, USA). Data are reported as the means and SD with percentages. The chi-square test and Student’s *t*-tests were performed in univariate analysis to determine the differences in parameters between the two groups. We proceed with the multivariate logistic regression analysis to identify the independent risk variables associated with 30 days mortality in TAAD. The *P* < 0.05 was considered statistically significant.

For the evaluation of survival Kaplan Meyer curves were created at the end of the analysis.

## Results

Demographic and clinical preoperative characteristics are presented in Table 1. The mean age of the patients was 66 ± 12.4 years with a range between 23 and 88 years.

**Table 1:** Preoperative and intraoperative characteristics

	Total (*n* = 340)	Diabetes (*n* = 34)	No diabetic (*n* = 306)	*P* value
Age ± SD	66 ± 12.4 (23–88)	72.1 ± 10.5 (46–85)	65.4 ± 12.4 (23–88)	0.001
Male	207 (60.9%)	12 (35.3%)	195 (63.7%)	0.001
Age >80 years	56 (16.5%)	11 (32.4%)	45 (14.7%)	0.008
Age >70 years	144 (42.4%)	21 (61.8%)	123 (40.2%)	0.016
Hypertension	241 (70,9%)	25 (73.5%)	216 (70.6%)	0.720
Smoke history	79 (23.2%)	6 (17.6%)	73 (23.9%)	0.416
COPD	41 (12.1%)	5 (14.7%)	36 (11.8%)	0.617
Dyslipidemia	57 (16.8%)	10 (29.4%)	47 (15.4%)	0.037
Previous cardiac surgery	27 (7.9%)	4 (11.8%)	23 (7.5%)	0.385
Cardiogenic shock	77 (22.6%)	4 (11.8%)	73 (23.9%)	0.110
Creatinine (mg/dl)	1.17 ± 0.7 (0.2–7)	1.15 ± 0.7 (0.5–4.1)	1.18 ± 0.7 (0.2–7)	0.370
EF %	53.1 ± 8.2 (20–75)	54.7 ± 9.2 (30–75)	53 ± 8.1 (20–75)	0.196
Anuria or oliguria <10 ml/h	68 (20%)	3 (8.8%)	65 (21.2%)	0.086
Cardiac massage	16 (4.7%)	3 (8.8%)	13 (4.2%)	0.232
Mechanical ventilation before arrival in operating room	82 (24.1%)	6 (17.6%)	76 (24.8%)	0.353
Inotropes	81 (23.8%)	5 (14.7%)	76 (24.8%)	0.188
Critical preoperative state^a^	123 (36.2%)	9 (26.5%)	114 (37.3%)	0.214
Tamponade	114 (33.5%)	14 (41.2%)	100 (33.7%)	0.319
Proximal surgery				
Ascending	226 (66.5%)	23 (67.6%)	203 (66.3%)	0.878
Ascending + valve	8 (2.4%)	1(2.9%)	7 (2.3%)	0.811
Bentall	101 (29.7%)	10 (29.4%)	91 (29.7%)	0.968
Valve sparing root replacement	2 (0.6%)	0	2 (0.7%)	0.636
Cabrol	3 (0.9%)	0	3 (1%)	0.562
Distal surgery				
Hemiarch	295 (86.8%)	29 (85.3%)	266 (86.9%)	0.790
Total arch replacement				
ET	38 (11.2%)	5 (14.7%)	33 (10.8%)	0.491
Frozen ET	7 (2.1%)	0	7 (2.1%)	0.373
Perfusion data				
Circulatory arrest ±DS (range)	33.4 ± 18.7 (0–110)	30.3–18.8 (0–77)	33.8 ± 18.7 (0–110)	0.340
Aortic crossclamping ±DS (range)	117.3 ± 48.7 (30–499)	126.8 ± 56.3 (55–299)	116.3 ± 47.7 (30–499)	0.378
CPB time ± DS (range)	206.2 ± 75.1 (45–701)	196.7 ± 70.7 (100–384)	207.2 ± 75.6 (45–701)	0.327
Minimal temperature	25.2 ± 3.4 (18–36.4)	26.1 ± 3.4 (20–33)	25.1 ± 3.4 (18–36.4)	0.108

a Critical preoperative state as defined by Euroscore II.

The average age of the patients with diabetes was statistically higher (72.1 ± 10.5) than the surviving patients (65.4 ± 12.4) (*P* = 0.001). The majority of patients in the diabetic group are female with a statistically significant difference compared to nonpeople with diabetes (64.7% vs. 32.3%, *P* = 0.001). Furthermore, among the preoperative variables, the percentage of dyslipidemic patients was higher in the diabetes group (*P* = 0.037).

At the time of admission, 36.2% of the sample had a preoperative critical state, without statistical differences in the two groups.

The different types of repairs performed, and the operating data are summarized in Table 1. Regarding our surgical approach we prefer the use of axillary artery cannulation [8] with a greater use of a ‘simple’ replacement of the supra-junctional portion of the aorta, associated with complete replacement of the aortic arch in 13.3% of patients. No statistically significant differences were found between the two groups of patients in terms of surgical procedure.

### Early outcomes

Perioperative outcomes such as bleeding requiring chest re-exploration, major stroke, lactate levels and ICU were comparable between the groups.

Postoperative complications are reported in Table 2.

**Table 2:** Complications

	Total (*n* = 307)	Diabete (*n* = 31)	No diabete (*n* = 276)	*P* value
Reopening	81 (26.5%)	8(25.8%)	73 (26.5%)	0.930
Arrhythmias (excluding AF)	38 (12.4%)	2 (6.5%)	36 (13.1%)	0.288
AF	105 (34.3%)	9 (29%)	96 (34.9%)	0.514
Pneumoniae	26 (8.5%)	3 (9.7%)	23 (8.4%)	0.804
Tracheostomy	54 (17.6%)	5 (16.1%)	49 (17.8%)	0.815
Coma	23 (7.5%)	3 (9.7%)	20 (7.3%)	0.630
Stroke	32 (10.5%)	3 (9.7%)	29 (10.5%)	0.881
Neurological damage discharge	35 (11.4%)	3 (9.7%)	32 (11.6%)	0.745
Cardiac arrest	22 (7.2%)	2 (6.5%)	20 (7.3%)	0.867
Mesenteric ischemia	12 (3.9%)	1 (3.2%)	11 (4%)	0.833
Sepsis	16 (5.2%)	1 (3.2%)	15 (5.5%)	0.597
Acute renal failure	49 (16%)	8 (25.8%)	41 (14.9%)	0.117
Length of respiratory support (hours)	186.5 ± 230.5 (1–1511)	243.5 ± 248.8 (8–961)	180 ± 228 (1–1511)	0.115
Lactate levels (mmol/l)	7.04 ± 3.82 (1.3–25.6)	7.48 ± 4.08 (2.6–19)	6.98 ± 3.79 (1.3–25.6)	0.438
Length of ICU stay (days)	11.7 ± 1(0.1–63.6)	14.9 ± 13.2 (0.8–47.6)	11.3 ± 11.8 (0.1–63.6)	0.265
Mortality 30 days				
Overall mortality	82 (24.1%)	12 (35.3%)	70 (22.9%)	0.098
Operating room	33 (9.7%)	3 (8.8%)	30 (9.8%)	0.218
ICU	49 (16%)	9 (29%)	40 (14.5%)	0.096

The primary outcome was 30-day mortality. Mortality data in the general sample showed 33 intraoperative deaths (9.7%) with a postoperative mortality during the ICU stay of 49 patients (16%). Although mortality turned out to be higher in the diabetic group (35.3%) than in the nondiabetic group (22.9%), it did not reach statistical significance (*P* = 0.098) (Table 3).

**Table 3:** Multivariate analysis

	OR	CI 95%	*P* value
Age >80	0.448	0.195–1.026	0.057
Hypertension	0.554	0.246–1.25	0.155
Smoking history	1.901	0.754–4.791	0.173
COPD	0.327	0.145–0.952	0.039
Dyslipidemia	0.468	0.19–1.15	0.098
Diabetes	0.417	0.159–1.098	0.077
Coronary artery disease	0.402	0.135–1.192	0.100
Coronary malperfusion	0.345	0.148–0.8	0.013
Cerebral malperfusion	1.207	0.482–3.024	0.687
Visceral malperfusion	0.411	0.157–1.077	0.070
Limb malperfusion	0.555	0.196–1.574	0.268
Tamponnade	0.728	0.355–1.493	0.386
Anuria or oliguria <10 ml/h	0.455	0.187–1.108	0.083
Mechanical ventilation before arrival in operating room	0.569	0.25–1.297	0.180

### Multivariate analysis

Based on the results obtained, we conducted a multivariate analysis with the aim of identifying the preoperative predictive variables of 30-day mortality.

The results obtained are shown in Table 3.

Diabetes was found not to be a statistically significant predictor value of in-hospital mortality.

### Long-term outcomes

**Fig. 1:**
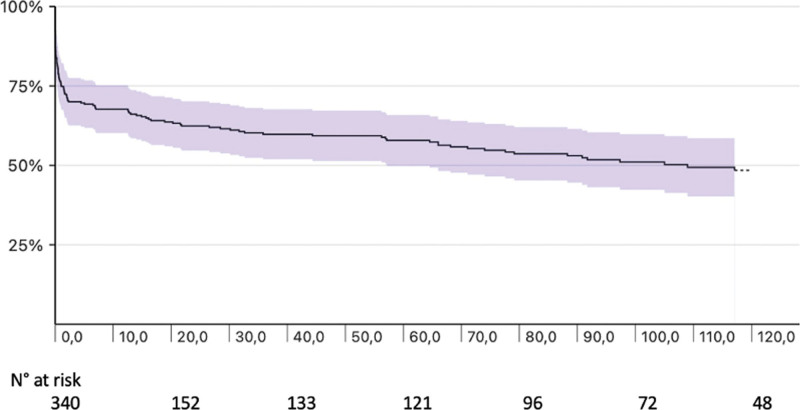
Kaplan–Meier curves showing long-term overall survival: 5 years 57.5% (95% CI: 51.7–63.2%), 10 years 48.3% (95% CI: 41.6–54.7%). CI, confidence interval.

The Kaplan Meyer curves for overall survival of the sample are shown in Fig. 1. Overall survival at 5 and 10 years was 57.5% (95% CI: 51.7–63.2%) and 48.3% (95% CI: 41.6–54.7%) respectively.

**Fig. 2:**
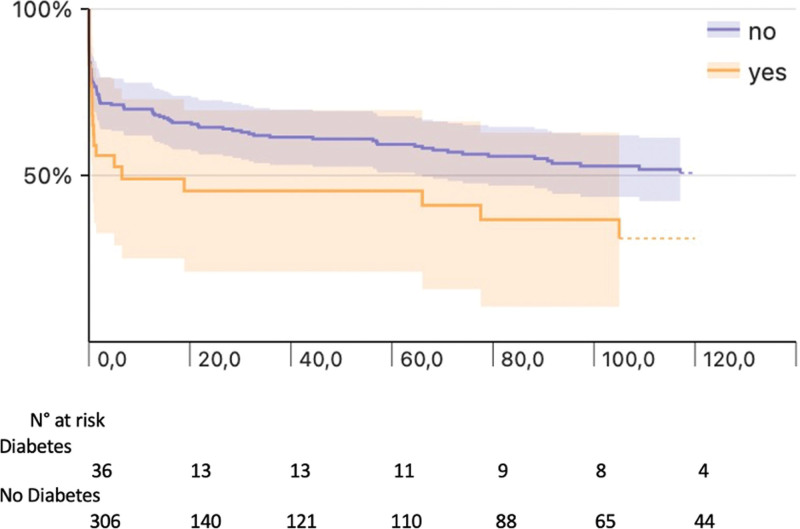
Kaplan–Meier curves showing long-term survival in the 2 groups: No = no diabetes: 5 years 59.1%, (95% CI: 52.8–64.9%) at 10 years 50.6% (95% CI: 43.4–57.3%). Yes = diabetes: survival 5 years 44.2% (95% CI: 26.6–60.5%) at 10 years 29.5% (95% CI: 13.2–47.9%). CI, confidence interval.

Analyzing the two study groups (Fig. 2), a better and statistically significant 10-year survival was observed in patients without diabetes (*P* = 0.024). Survival in the diabetes group was 44.2%, (95% CI: 26.6–60.5%) and 29.5% (95% CI: 13.2–47.9%) at 5 and 10 years respectively. Survival for no diabetes group was 59.1% (95%CI: 52.8–64.9%) at 5 years and 50.6% (95% CI: 43.4–57.3%) at 10 years.

## Discussion

Thanks to advances in diabetes care, the incidence of CVD in diabetic patients has decreased over the last decade. However, CVD are the leading cause of death in patients with DM and the risk of CVD in patients with DM is more than double that of patients without DM [9]. However, the relationship between diabetes and aortic dissection is still not completely clear [10,11].

There are several factors that might contribute to their association. First, diabetes is a well-established risk factor for atherosclerosis and hypertension. These conditions can weaken the walls of the aorta and increase the risk of aortic dissection [12,13]. As shown in our study, the presence of dyslipidemia is statistically higher in diabetic patients than in nondiabetic patients. However, we did not find differences in the percentage of hypertensive patients in the two groups.

Furthermore, chronic inflammation and oxidative stress are known to contribute to the development of vascular diseases, including aortic dissection. In the broader context of diabetes and its complications, chronic inflammation is generally considered detrimental: hyperglycemia can lead to increased production of pro-inflammatory molecules, triggering inflammation and the insulin resistance is associated with increased production of inflammatory cytokines by adipose tissue.

However, while the association between diabetes and aneurysms is complex and not fully understood yet, there is some evidence suggesting that diabetes may have a protective effect against the development of aortic aneurysms [14].

According to Nienaber [15], macrophages which produce inflammatory cytokines and matrix digesting metalloproteinases (MMPs) found in the aortic wall, could play a role in the aneurysmal evolution of the aorta by promoting vessel remodeling.

Moreover, chronic hyperglycemia in diabetes can lead to a process called glycation, where glucose molecules attach to proteins, altering their structure and function. This process may affect the integrity and strength of the aortic wall, making it more susceptible to dissection. However according to some authors [16,17] the glycated monomeric collagen can influence the secretion of MMP, reducing the level of MMP-9, MMP-2 and interleukin-6 by activated monocytes, thus inducing cross-links that may enhance the structural integrity of the arterial wall.

This observation, that glycated extracellular matrix inhibits monocyte MMP production, may conceptually lead to potential protection of DM against aortic growth and TAAD by stabilizing the extracellular matrix [18–20]. In 2018 Avdic *et al.* [21]. investigated the relationship between diabetes, aortic aneurysm and acute aortic dissection. The researchers analyzed data from a large cohort of patients and found that diabetes had significantly reduced risks of aortic aneurysms and aortic dissection as reduced risk of mortality after hospitalization for aortic aneurysm, compared to control subjects. For the authors the cause of this seems to be related to the protective role of glycated cross-links in aortic tissue in the progression of aortic diseases.

Several studies have examined the association between diabetes and aortic dissection.

Guo *et al*. [22] recently reported an association of mutations in the smooth muscle α-actin gene *(ACTA2*) with thoracic aortic disease, stroke and coronary artery disease (CAD), with the latter clearly and causally related to type 2 DM. However, in our study sample we did not see a statistically significant difference in the rate of CAD in patients with (8,8%, three patients) or without diabetes (9.8%, 30 patients) (*P* = 0.855).

Prakash *et al*. [23], in a national case-control study, found that diabetes is associated with a decreased rate of hospitalization due to TAAD in proportion to nondiabetic patients. The researchers also suggested a potential direct effect of hyperglycemia on the aortic wall to inhibit/reduce dissection progression.

The authors found significant positive association between diabetes and chronic ischemic heart disease. We didn’t find any correlation between the two groups in terms of CAD (three patients in diabetic group and 30 patients in nondiabetic group *P* = 0.855).

While these mechanisms suggest a potential protective factor between glycation and aortic dissection, it’s important to note that the research in this area is still evolving, and the precise role of glycation in aortic dissection requires further investigation. Other factors, such as hypertension, genetic predisposition, and connective tissue disorders, such as Marfan and Ehlers-Danlos syndrome, also play significant roles in the development of aortic dissection [24].

However, in literature there are no evidence that people with diabetes have an increased prevalence of connective tissue abnormalities.

Although most studies agree on the protective role of diabetes in the comparison of aneurysms, there is no univocal opinion regarding its association in aortic dissection.

A recent study by Zheng *et al.* [7] showed that insulin resistance induces the phenotypic switching of vascular smooth muscle cells from contractile to synthetic, which contributes to the occurrence of TAAD.

Our study did not report a negative effect on in-hospital mortality among patients with diabetes, not even in multivariate analysis [1,23,25,26].

Furthermore, our results are encouraging and confirmed by others works in literature.

Jiménez-Trujillo *et al*. [1] reported that mortality was significantly lower among diabetic patients discharged after hospitalization for thoracic aortic aneurysm and dissection. However, they also analyzed patients with chronic aneurysms not operated in an emergency/urgency and who underwent a ‘simple’ endovascular treatment.

A recent population-based cohort study of Japanese residents of Koba *et al*. [25] reported that diabetes was inversely associated with mortality from total aortic diseases compared to other factors such as smoking habits, hypertension and dyslipidemia.

He *et al.* [26], in a case-control study conducted to evaluate the variability between aortic dissection risk and diabetes, also concluded that diabetes was estimated to be associated with a reduced risk of dissection. Moreover, the authors reported no significant differences in in-hospital mortality when comparing diabetic and nondiabetic patients as in our series.

A recent meta-analysis by Li *et al.* [27] reported a lower risk of aortic dissection in patients with DM.

However, we must emphasize that 10-year mortality in diabetic patients was statistically higher than in the nondiabetic group. This could also be explained by two factors, the first linked to age. In our sample the patients in the nondiabetic group are younger. Secondly, diabetic patients may have an increased risk of mortality related to diabetes complications.

### Limitations

We must acknowledge some limitations of our study, which derive mainly from the limited sample of patients, the large study period and the retrospective nature of this study.

Furthermore, we did not classify diabetic patients according to the type of treatment used for blood glucose control and the degree of blood glucose control before or after surgery.

### Conclusions

In conclusion this study found that patients with DM have the same risk of in-hospital mortality as patients without diabetes. However, the relationship between diabetes and TAAD is complex, and additional research is needed to fully understand the mechanisms involved. Shedding light on this relationship may possibly provide new insight into the causes, prevention and treatment of AD.

In the era of modern diagnostic tools and endovascular treatments [28–31] the evaluation of each risk factor is important to guarantee a better prognosis of the aortic dissection, which remains lethal to this day.

This study was reviewed and approved by the Ethics Committee of Sperimentazione clinica della Toscana, AREA VASTA SUD EST (n° 23547). The Ethics Committee waived the need of written informed consent to participate in this study.

## Acknowledgements

Considering the retrospective nature of this study, the Institutional Ethics Review Board (TAAD23547) has given its approval, without the need to have the written informed consent.

### Conflicts of interest

There are no conflicts of interest.
